# Clinical significance of STEAP1 extracellular vesicles in prostate cancer

**DOI:** 10.1038/s41391-021-00319-2

**Published:** 2021-02-15

**Authors:** Karan Khanna, Nikki Salmond, Kalan S. Lynn, Hon S. Leong, Karla C. Williams

**Affiliations:** 1 Faculty of Pharmaceutical Sciences, The University of British Columbia, Vancouver, Canada; 2 Lawson Health Research Institute, London, ON Canada; 3 Translational Urology Research Laboratory, Department of Medical Biophysics, Faculty of Medicine, University of Toronto, Toronto, ON Canada; 4 Biological Sciences Platform, Sunnybrook Research Institute, Toronto, ON Canada

**Keywords:** Diagnostic markers, Cancer screening

## Abstract

**Background:**

Extracellular vesicles (EVs) are cell-derived lipid bilayer enclosed structures shed from the plasma membrane by all cell types. Evidence of EV presence in biological fluids has led to considerable efforts focused on identifying their cargo and determining their utility as a non-invasive diagnostic platform for cancer. In this study, we identify circulating STEAP1 (six-transmembrane epithelial antigen of the prostate 1)-positive EVs in the plasma of healthy males and prostate cancer patients and evaluate its diagnostic and prognostic significance.

**Methods:**

STEAP1 was identified on EVs in prostate cancer patient plasma. EVs were validated using electron microscopy, Western blot, nanoparticle tracking analysis, and nanoscale flow cytometry. STEAP1-positive EVs were quantified for 121 males with prostate cancer and 55 healthy age-matched control males. An evaluation of STEAP1 in prostate cancer tissue was also performed using established prostate cancer cohort data (TCGA, MSKCC, and SU2C/PCF Dream Team).

**Results:**

Evaluation of STEAP1-positive EVs by nanoscale flow cytometry identified a significant increase in prostate cancer patient plasma compared to healthy males. However, no association was found between total STEAP1 EV levels and disease recurrence or overall survival. Cohort data from prostate cancer tissue also found STEAP1 to be elevated in prostate cancer while no significant association with recurrence or overall survival was identified.

**Conclusions:**

STEAP1 is known to be enriched on the cells of the prostate with potential clinical significance in prostate cancer. Our results identify and quantitate STEAP1-positive EVs in plasma and provide rationale for a STEAP1 EV-based liquid biopsy as a diagnostic strategy in prostate cancer.

## Introduction

Prostate cancer (PCa) is the most commonly diagnosed non-cutaneous cancer in males. Screening for PCa using the prostate-specific antigen test (PSA) has reduced the incidence of late-stage PCa and PCa mortality [1]. However, it is also associated with an increase in the detection of benign and non-cancerous lesions [1, 2]. When PSA levels are high (>10 ng/mL), 67% of individuals are likely to have a PCa diagnosis confirmed by biopsy, whereas a PSA of 4–10 ng/mL results in a positive biopsy diagnosis in only 30–35% of patients [2]. Thus, PSA testing can incorrectly diagnose PCa in otherwise healthy males [3]. This can lead to a significant overdiagnosis of PCa which results in unnecessary needle core biopsies [4]. Early detection of PCa is important as it can mitigate the risk of disease progression associated with late-stage detection. Accordingly, the identification of non-invasive biomarkers that are capable of increasing the accuracy of PCa screening is of great interest for improving the clinical management and long-term survival of PCa patients.

Extracellular vesicles (EVs) have garnered substantial interest as a diagnostic and prognostic tool in cancer [5, 6]. EVs are nanosized lipid enclosed membrane vesicles (~50 to >1000 nm) released by most cell types, including cancer cells. Proteins, nucleic acids, lipids, and glycans are packaged into EVs and can reflect the general composition of their cell of origin [7]. EVs are involved in cell-to-cell communication and can deliver their functional cargoes to recipient cells at local and distant sites to influence physiological and pathological processes [8, 9]. As EVs are readily detectable in serum, plasma, urine, and saliva, their potential as a non-invasive platform in the health and disease monitoring is well-recognized and has been investigated by many groups [10–15].

The metalloreductase STEAP1 (six-transmembrane epithelial antigen of the prostate 1) is a transmembrane protein that has enhanced expression in prostate tissue [16]. STEAP1 is readily expressed in prostate tumors and elevated in all stages of the disease [16]. Given its restricted expression in normal human tissues, STEAP1 has been proposed as a target for immunotherapy and for imaging of PCa [16–18]. Here, we isolated EVs and identified the presence of STEAP1-positive EVs in the plasma of healthy males and males with PCa. We then performed nanoscale flow cytometry analysis of STEAP1 EV levels in whole plasma using 121 PCa patients and 55 age-matched controls. Our results demonstrate that elevated STEAP1-positive EV levels in plasma are significantly associated with a PCa diagnosis.

## Materials and methods

### Blood collection

Blood samples were obtained from 121 PCa patients (age range 50–80-years old) and 55 age range matched (age range 50–70-years old) healthy males (Supp. Table 1 for PCa clinical characteristics). For the PCa cohort, blood was collected prior to needle core biopsy using a 10 ml K2-EDTA collection tube and centrifuged at 2500 × *g* for 15 min. The plasma layer was removed and stored in liquid nitrogen until shipment. Samples were shipped on dry ice, thawed, mixed, and aliquoted. Pathological evaluation of needle core biopsy was performed on men with suspected PCa and all men used in this study had a histopathological diagnosis of PCa encompassing Gleason Group 1–5. All individuals provided informed consent in accordance with institution protocol and study approval was obtained by the institutional review board of UBC (IRB#H17-01442). Healthy blood samples were collected by Innovative Research Inc. using blood collection bags. Four hundred and fifty milliliters of blood plus K2-EDTA was centrifuged at 5000 × *g* for 15 min. The plasma was removed, aliquoted, and shipped on dry ice. Once received, samples were randomly assigned a number. This study is compliant with all relevant ethical regulations on the use of human plasma.

### EV isolation from plasma via size-exclusion chromatography

One milliliter plasma was thawed on ice and filtered using a 0.8 µm filter. An IZON qEVoriginal/70 nm size-exclusion chromatography (SEC) column (IZON Sciences Ltd.) was brought to room temperature and equilibrated with two-column volumes (20 ml) of 0.2 µm filtered PBS. Subsequently, 500 µl of plasma was applied to the top of the column, and the flow-through was collected immediately. The flow of the sample through the column was maintained by continuously adding 2 ml 0.2 µm filtered PBS, to ensure the column was not allowed to run dry. After 3 ml flow-through was collected, a 2.5 ml EV fraction was collected as well as four subsequent 1 ml fractions (fractions 1–5). Isolated EVs and fractions were concentrated to 100 µl using a 10 kDa molecular weight cut off regenerated cellulose membrane Amicon® Ultra-4 centrifugal concentrator (Millipiore, Sigma).

### Protein concentration quantification

The protein concentration of purified EVs and subsequent fractions were determined using Pierce™ Rapid Gold BCA Protein Assay Kit (Thermo Fisher Scientific) according to the manufacturer’s instructions.

### Nanoparticle tracking analysis

The concentration and size of particles present in the isolated fractions were analyzed using nanoparticle tracking analysis. Samples were diluted 1:1000/2000 in 0.2 µm filtered PBS (Wisent) and particles analyzed using the NanoSight LM10 (Malvern PANalytical) with a 488 nm blue laser. A syringe pump was used to create a continuous flow of sample through the chamber at speed 40, and three 30 s videos were acquired at camera level 14. The resultant data were analyzed at detection threshold 5 using NTA software version 3.2.16.

### Electron microscopy

Isolated fractions were fixed in 2% electron microscope grade paraformaldehyde (Fisher Scientific) in PBS and adsorbed onto formvar/carbon-coated 200 mesh nickel grids for ~1 min. Grids were negatively stained by incubation with pre-filtered 1% uranyl acetate (Fisher Scientific) pH 4.6 for 30 s. Grids were blotted dry before being imaged using a Helios NanoLab 650, fitted with a STEM detector, (Thermofisher, Systems for Research, Kanata, ON, Canada) in scanning transmission bright field imaging mode at 30 KV.

### Western Blot

Ten or twenty micrograms of each SEC isolated fraction was mixed with 10% reducing buffer (10 x Novex Bolt™) and 25% loading buffer (Novex Life Technologies) followed by heating at 95 °C for 10 min. Reducing buffer was not added to the sample if non-reducing conditions were required for CD63 detection. Samples were loaded onto Bolt™ 4–12% Bis-Tris Plus gradient Gels (Thermo Fisher Scientific) and Precision Plus Protein™ Kaleidoscope Ladder (BioRad) was used as a molecular weight marker. Gel was ran in MOPS running buffer (50 mM MOPS (Sigma), 50 mM Tris Base, 0.1% SDS, 1 mM EDTA, pH 7.7 at 200 V for 30 min. All reagents from Fisher Bioreagents unless otherwise stated). Proteins were transferred from the gel onto 0.45 µm nitrocellulose membrane (BioRad) using wet transfer at 30 V for 1.5 h (transfer buffer: 190 mM glycine, 25 mM Tris Base). The membrane was blocked in 5% milk dissolved in TBS-T (20 mM Tris base, 160 mM NaCl, 0.1% Tween) for 1 h and then incubated overnight with primary antibodies in 1% milk in TBS-T at 4 °C. The membrane was washed three times with TBS-T for 10 min and then incubated with secondary Li-COR IRDye® 680RD or IRDye 800CW antibodies in 1% milk in TBS-T for 1 h at room temperature. After repeating the washing as described for the primary antibody, the membrane was washed with MilliQ water and imaged on the Li-COR Odyssey® CLx using Image Studio Lite software 5.2.5. More information on antibodies used can be found in Supplementary Table 2.

### Nanoscale flow cytometry of EVs

Nanoscale flow cytometry analysis of EVs was performed as we have previously described [19]. Briefly, ten microliters of whole plasma or isolated SEC fraction was incubated with fluorescently labeled antibodies for 30 min at room temperature in the dark. For details of antibodies used, see Supplementary Table 2. Following incubation, the sample was diluted by re-suspension in 300 µL of 0.02 µm filtered PBS and each sample was transferred into a 96-well plate. To prevent cross-contamination between samples, a PBS wash was done between each sample, and at the end of each row, a 1% Contrad wash was followed by a water wash. Samples were analyzed on the CytoFLEX S (Beckman Coulter) instrument for 30 s at a slow speed of 10 µL/min in water using 405 nm violet side scatter trigger. The Violet side scatters detection threshold was set at 1027. Gain settings used: FITC – 500. BV421 - 77. APC - 533. PE – 135. Isotype controls were used to guide manual gating of populations of interest using CytoFLEX CytExpert 2.3 software. Data were exported to Excel and the total number of events that occurred within the gate during the 30 s measurement was used for statistical analysis in GraphPad Prism 8.

### Lysis of EVs

EVs were lysed before running on the CytoFLEX S (Beckman) by mixing the plasma/fractions incubated with antibody in 300 µl 0.5% SDS in MilliQ water or 1% Triton-X 100.

### Analysis of cancer genomics study data

RNA sequencing data were accessed through cBioPortal [20]. Data were accessed from the following: TCGA [21], MSKCC [22], and Metastatic Prostate Adenocarcinoma SU2C/PCF Dream Team [23]. Clinical Gleason Category data and STEAP1 mRNA expression Z-Scores (RNA Seq V2 RSEM) (log2) (*n* = 290) and was downloaded and analysed by Gleason Score [21]. Progression status, alive/deceased status, and respective STEAP1 mRNA Expression, RSEM values (Batch normalized from Illumina HiSeq_RNASeqV2) (log2) (*n* = 488) were downloaded and analysed based on status [21]. PCa STEAP1 mRNA expression (*Z*-Scores) compared to expression in normal prostate sample data were downloaded and analysed based on Radical Prostatectomy Gleason Score (*n* = 111) and progression status (disease free or recurred) (*n* = 112) [22]. STEAP1 mRNA expression values (FPKM polyA) (log2) from an advanced PCa cohort were downloaded and analysed based on overall survival status (*n* = 81) [23].

### Software, statistical analysis, and data acquisition

Nanoscale flow cytometry data and images were obtained from CytoFLEX CytExpert 2.3 software. Each healthy and PCa patient sample was analyzed twice in independent experiments, performed on different days, to ensure reproducibility of the technology. The average of two runs was used for statistical analysis. Sample analysis and data collection were performed blinded to clinical data. Image Studio 2.5.2 was used to visualize the Western blots imaged on the Li-COR Odyssey® CLx. Adobe Illustrator 24.1.2 was used to prepare the figures. Representative images are shown and Western blot for each marker was performed at a minimum of *n* **=** 3. The collected data were handled in Microsoft Excel and processed using GraphPad Prism 8.4.2. The receiver operator characteristics curve was generated using Wilson/Brown method on Prism with a 95% confidence interval. The distribution of collected data was analyzed for normality using Shapiro-Wilk. Data that was proven to normally distributed were analyzed using parametric students' *T*-test, ordinary one-way ANOVA, or paired *T*-test. Data that were not normally distributed were analyzed using non-parametric Mann–Whitney *t*-test, Kruskal–Wallis ANOVA, or Wilcoxon matched-pairs signed-rank test. The correlation was assessed using simple linear regression. *p **<** 0.05. **p **<** 0.01. ***p **<** 0.001. ****p **<** 0.0001.

## Results

### Circulating STEAP1 EVs are identified in plasma

**Fig. 1:**
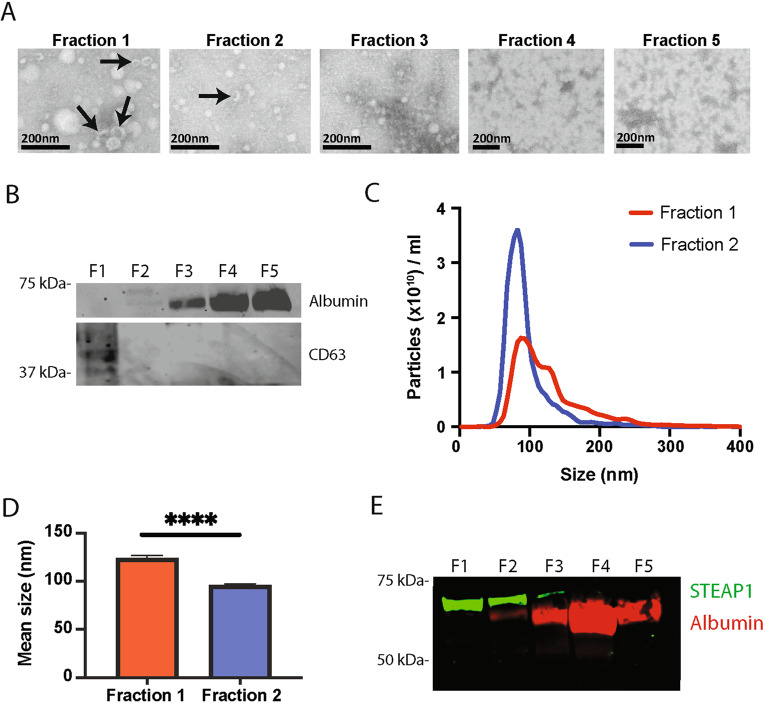
STEAP1 prostate derived EVs can be isolated from plasma using SEC. **A** SEC was used to isolate EV fractions from PCa patient plasma. Fractions were fixed in 2% paraformaldehyde, adsorbed onto Formvar/carbon-coated nickel 200 mesh grids and negatively stained using 1% uranyl acetate. The images were acquired using a Helios NanoLab 650 in scanning transmission mode at 30 kV. *n* = 2. **B** 10 µg of isolated fractions were Western blotted for expression of EV marker CD63 and EV preparation contaminant protein albumin. *n* = 3. **C**–**D** EVs were isolated from PCa plasma by SEC, the size and concentration of particles present in EV fractions 1 and 2 were analyzed by nanoparticle tracking analysis. *n* = 12. Unpaired *t*-test. ****p < 0.0001. **E** Western blot of 10 µg of isolated fractions for prostate marker STEAP1 and EV contaminant protein albumin. *n* = 3.

As EVs carry proteins representative of their cell of origin and STEAP1 expression has been reported to be elevated in PCa relative to healthy prostate tissue, this elevated expression of STEAP1 may be reflected by EVs present in biological fluids such as blood. To determine if STEAP1 could be detected on circulating EVs in the blood, EVs were isolated from the plasma of individuals with a biopsy confirmed diagnosis of PCa and assessed for STEAP1 expression (Fig. 1). SEC was used to isolate EVs from the plasma of PCa patients and isolated factions were analysed for EVs using scanning transmission electron microscope (STEM), Western blot, and nanoparticle tracking analysis (Fig. 1A–E). EVs with a cup-shaped morphology were identified in fractions 1 and 2 (Fig. 1A). EV isolation was further validated by Western blot for the EV marker CD63 which was shown to be abundant in fraction 1. EV fractions 1 and 2 are also shown to be devoid of contaminating protein albumin. Albumin was abundant in the later more proteinaceous non-EV fractions 3–5 (Fig. 1B). Finally, nanoparticle tracking analysis showed that particles with an average size of 123.2 and 95.01 nm were present in EV fractions 1 and 2 respectively (Fig. 1C and D). Analysis of STEAP1 expression in isolated fractions by Western blot identified STEAP1 in EV fractions 1 and 2, with low to negligible levels in later protein-rich, non-EV, fractions (Fig. 1E).

**Fig. 2:**
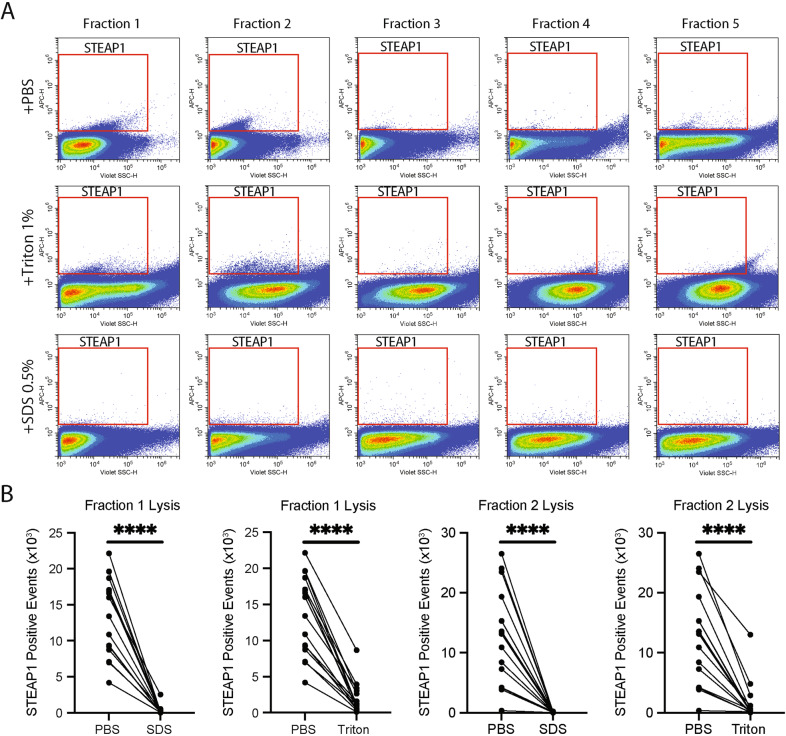
Isolated PCa plasma-derived STEAP1 EVs can be detected by nanoscale flow cytometry. **A** Representative images of STEAP1 populations as detected by nanoscale flow cytometry in EV fractions isolated from PCa plasma by SEC. EVs were either un-treated, or treated with 1% Triton™ X-100 or 0.5% SDS. **B** The EV fraction 1 and subsequent fraction 2 was analyzed for STEAP1 in the absence and presence of 1% Triton™ X-100 or SDS 0.5%. Healthy: four samples. PCa: 12 samples. Mann–Whitney test and unpaired *t*-test. ****p < 0.0001.

In order to quantitate the number of circulating STEAP1 positive EVs in plasma, nanoscale flow cytometry was employed. Nanoscale flow cytometry can be used to detect and quantify nanosized particles between ~80 nm and 1 µm at single event resolution [15, 19, 24]. EVs derived from cell culture, plasma, and serum have been successfully analysed using various nanoscale flow cytometry platforms [15, 25–28]. Here, SEC isolated fractions 1–5 were analysed for STEAP1 by nanoscale flow cytometry. STEAP1-positive events were identified in EV fractions 1 and 2 but not in the later non-EV fractions 3–5 (Fig. 2A). To determine if these populations were bonafide EVs, fractions 1 and 2 were treated with detergent to lyse EVs [29]. Treatment with 1% Triton™ X-100 or 0.5% SDS reduced the mean number of STEAP1-positive events in fractions 1 (by 85.7% and 97.7%, respectively, *p* < 0.0001) and in fraction 2 (87.8% and 99.2%, respectively, *p* < 0.0001) (Fig. 2A and B). Overall, these data demonstrate that circulating STEAP1-positive EVs are present in the plasma of PCa patients and can be enumerated through nanoscale flow cytometry.

### Elevated STEAP1 EV levels associate with PCa

**Fig. 3:**
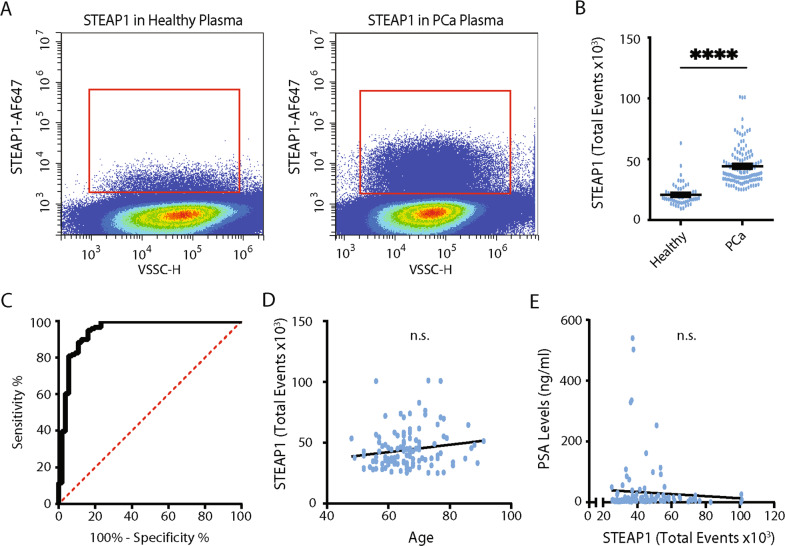
STEAP1 EVs can be detected in whole, unpurified plasma using nanoscale flow cytometry. **A** Representative images of STEAP1 populations in the plasma of healthy individuals and PCa patients as detected by nanoscale flow cytometry. **B** The number of STEAP1 positive events, excluding any the STEAP1-positive platelet events, in 55 healthy and 121 prostate cancer plasma samples was quantified using nanoscale flow cytometry. Each sample was analyzed twice in independent repeat experiments, the average of the two experiments was used for analysis. +SEM. Mann–Whitney test. ****P < 0.0001. **C** A receiver operating characteristic curve was generated using Graph Prism 8 to illustrate the diagnostic capabilities of STEAP1 positive events to distinguish between healthy males and males with PCa. **D**, **E** The correlation between the number of STEAP1 positive events in plasma and age (*n* = 121) (**D**) and PSA levels (*n* = 121) (**E**) was analyzed using linear regression.

Following identification and characterization of STEAP1-positive EVs from SEC isolated fractions of plasma, whole, unpurified, plasma from heathy (*n* = 55) and PCa subjects (*n* = 121) was analysed for STEAP1-positive EVs by nanoscale flow cytometry. STEAP1-positive events were detected by nanoscale flow cytometry in both healthy and PCa patient plasma (Fig. 3A). It has been reported that platelets and platelet-derived EVs are abundant in plasma and are detected by nanoscale flow cytometry [19, 24, 30, 31]. Importantly, as previous work has demonstrated that particles in a plasma can non-specifically associate or aggregate with platelet and EV membranes, to ensure that our identified STEAP1-positive EVs were not associated with platelets and platelet-derived EVs we analyzed 10 healthy and 10 PCa plasma samples for platelet specific markers CD9, CD41, and CD42a (Supp. Fig. 1). Higher platelet levels in healthy individuals were noted and are likely attributed to differences in blood collection protocols [19]. STEAP1 EVs were low or negative for platelet markers (Supp. Fig. 1D). Interestingly, we did note that some CD9 platelets were STEAP1 positive suggesting that STEAP1 EVs were possibly sticking to platelets, therefore the CD9-STEAP1 positive platelet population was excluded from all subsequent STEAP1 analysis (Supp. Fig. 1E). STEAP1 and CD9-positive populations relative to isotype controls, and reproducibility between runs, are shown in Supp. Fig. 2.

Next, the number of STEAP1 positive events were quantified in plasma from patients with histologically confirmed PCa (*n* = 121) and healthy, age-matched (50–70 year old), male donors (*n* = 55). Patient data are shown in Supplementary Table 1. STEAP1 EV levels were found to be significantly elevated in men with PCa compared to healthy males (44,230 ± 15,701 and 20,659 ± 9132 total events, respectively, *p* < 0.0001) (Fig. 3B). A comparison of patients with PCa to healthy male donors by a receiver operating characteristic (ROC) curve shows an AUC of 0.95 (95% CI: 0.90–0.99) with a Specificity of 76.79% (CI: 64.23–85.90%) when Sensitivity is set at 100% (95%CI: 96.9–100.0%) (Fig. 3C). Our analysis of STEAP1-positive EVs excluded platelet-associated STEAP1-positive events, which likely result from a platelet-EV association. To assess what impact this had on our analysis we also examined STEAP1 levels without any exclusion parameters. Similar results were found although we did find a slight reduction in AUC (0.93; 95% CI:0.89–0.98) (Supp. Fig. 3). A known issue of PSA testing is its association with age [32]. To determine if STEAP1 levels were associated with age or PSA a correlation analysis was performed. No significant correlation was found between STEAP1 levels and age or PSA (Fig. 3D and E). Overall, this supports the utility of STEAP1 as a biomarker for PCa screening to improve true-positive detection rates.

### STEAP1 levels in PCa risk stratification and prognostication

**Fig. 4:**
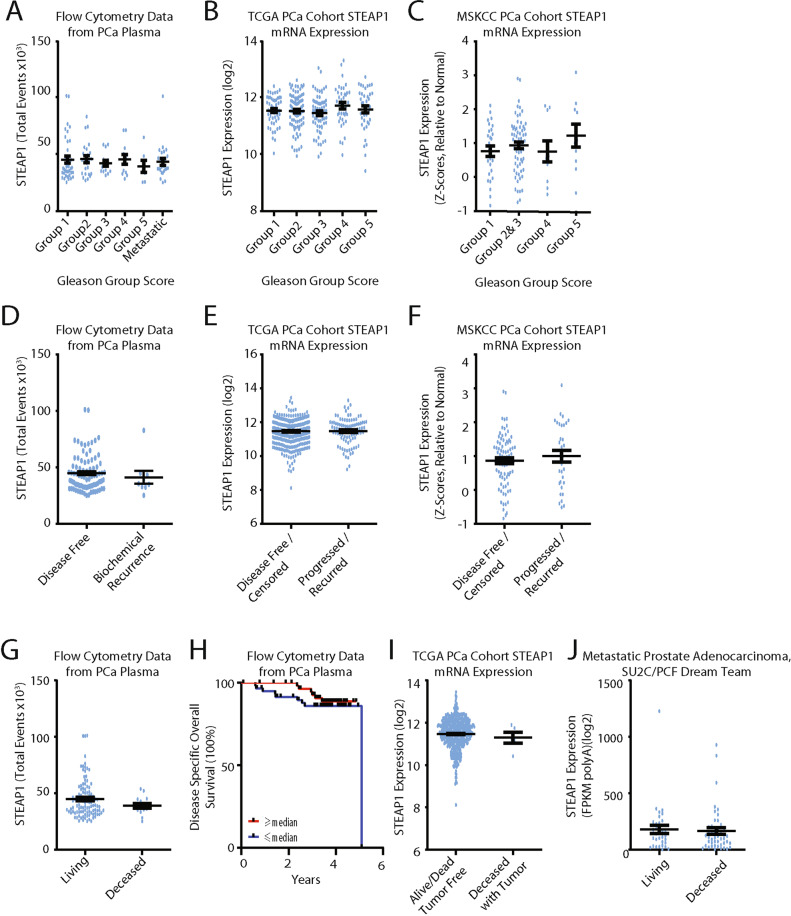
Quantification of STEAP1 EVs does not risk-stratify patients or provide prognostic value. **A** Analysis of STEAP1 positive events in the plasma of PCa patients based on Gleason Group score. Kruskal–Wallis ANOVA with multiple comparisons. *n* = 121. ±SEM, Tukey’s test. **B**, **C** STEAP1 mRNA levels from TCGA (*n* = 290) and MSKCC (*n* = 111) prostate cancer cohorts were assessed for STEAP1 expression between the diagnosed Gleason Group score. ±SEM, Tukey’s test. **D** STEAP1 positive events in whole plasma of PCa patients that remained disease free and those that had a biochemical recurrence. Unpaired *t*-test + SEM. **E**, **F** STEAP1 mRNA levels from TCGA (*n* = 488) and MSKCC (*n* = 111) prostate cancer cohorts were compared for STEAP1 expression between the disease-free individuals and individuals whose disease recurred. Unpaired *t*-test + SEM. **G**, **H** Analysis of STEAP1 positive events in the plasma of PCa patients based on survival status (**G**) and overall survival (**H**). Overall Survival curve comparison: Log-rank (Mantel-Cox) test. **I**, **J** STEAP1 tissue expression levels based on survival status. Unpaired *t*-test, +SEM.

To determine if STEAP1 levels associated with disease severity, STEAP1 levels were analyzed by Gleason Group. Individuals with an initial diagnosis of metastatic disease were grouped into a metastatic category for analysis. The majority of metastatic patients, 75%, were diagnosed with Gleason Group 5 disease (Supplementary Table 1). Gleason Group 1–5, and individuals with metastatic disease all had similar STEAP1-positive EV levels, and no significant differences were found across any of the groups (Fig. 4A).

STEAP1 expression has been reported in multiple studies to be elevated in PCa relative to a healthy prostate [33–36]. However, the extent to which it risks stratifies or prognosticates is uncertain. There are reports demonstrating PCa stratification and prognostication using STEAP1 protein or RNA expression levels [34, 35], while other reports find no such association [33, 36]. Potentially our EV analysis does not have the necessary sensitivity to distinguish between Gleason Groups. To further evaluate STEAP1 expression levels in PCa, RNA expression levels were evaluated using publically available data from the TCGA primary prostate cancer adenocarcinoma cohort [21] and MSKCC prostate cancer adenocarcinoma cohort [22]. STEAP1 mRNA levels for individuals with histologically reviewed Gleason scores (TCGA, *n* = 290; MSKCC, *n* = 111) were assessed and no significant differences in STEAP1 expression were found between any of the Gleason Groups (Fig. 4B and C).

While elevated STEAP1 EV levels were found to be diagnostic for PCa, total STEAP1 EV counts could not provide prognostic information. To further evaluate STEAP1 as a potential prognostic marker, analysis of STEAP1 EV levels in patients with a biochemical recurrence was performed. 7.5% of our patient cohort had a biochemical recurrence (either local or metastatic) during the 1–5-year clinical follow-up period. No significant difference was identified between disease-free individuals and individuals whose disease recurred (Fig. 4D). Next, an evaluation of STEAP1 RNA expression levels was performed using the primary prostate cancer adenocarcinoma cohort TCGA, PanCancer [37], and MSKCC prostate cancer adenocarcinoma cohort [22]. No significant differences were identified between individuals with a disease-free status and individuals whose disease recurred for either the TCGA, PanCancer (*n* = 488), or MSKCC cohort (*n* = 111) (Fig. 4E and F). Next, an analysis of overall survival relative to STEAP1 EV levels and tissue mRNA expression was performed. No significant differences were identified for STEAP1-positive EV levels or mRNA levels based on patient overall survival status (Fig. 4G–J). Thus, while STEAP expression is clearly elevated in PCa tissue and our findings identify an elevated level of STEAP1-positive EVs in PCa, we did not find STEAP1-positive EVs capable of risk-stratifying or prognosticating patients.

## Discussion

STEAP1 expression is elevated in PCa, and here we show that STEAP1-positive EVs can be detected in plasma and are elevated in PCa. As a non-invasive screening tool, STEAP1-positive EV levels may represent a superior screening method over the PSA test.

The gold standard for diagnosing PCa is a tissue biopsy. This process can result in several challenges, and a recent study identified that most of the costs associated with a prostate biopsy occurred from complications that arose in the days following the biopsy [38, 39]. Unnecessary biopsies are often a result of PSA screening as the PSA blood test carries a high false-positive rate leading to biopsies in otherwise healthy individuals, psychological stress, and excessive healthcare costs [4]. Our work demonstrates that elevated levels of STEAP1 EVs are significantly associated with a PCa diagnosis, suggesting that quantitation of STEAP1 EVs could be used as a screening platform to improve the selection of individuals for follow-up biopsy.

While STEAP1 appears to have clear diagnostic capabilities, whether STEAP1 has utility in risk-stratifying or prognosticating patients is less well described. Our work suggests that total STEAP1 EV levels have utility as a diagnostic but do not appear to risk-stratify or prognosticate. To further evaluate the prognostic capabilities of STEAP1, we used publically available data (TCGA, MSKCC, SU2C/PCF Dream Team) and analysed STEAP1 mRNA expression across Gleason Groups and in relation to patient outcome. STEAP1 mRNA expression was not capable of risk-stratifying or prognostication. This is also consistent with two other reports [33, 36] demonstrating an inability of STEAP1 expression to differentiate between Gleason Scores or patient outcomes.

Recently STEAP1 was found to be expressed on PCa cell culture-derived EVs and, in-line with our work, abundance was not found to indicate disease aggressiveness [40]. It is however important to note that while we did not find prognostic significance from an evaluation of total STEAP1 EVs there may be sub-populations of STEAP1 EVs with predictive or prognostic capabilities. Of interest, an elevated count of large EpCAM positive tumor cell fragments in the blood of PCa patients is associated with poor patient outcomes [41] and, large EVs, termed oncosomes, released by PCa cells are associated with advanced disease [42–44]. It is possible that distinct populations of STEAP1 EVs identified based on size or EpCAM status could have prognostic significance, but this has yet to be determined. In addition, it could be of interest to examine the expression of other markers on STEAP1 EVs. For instance, elevated expression of GLUT1 [45] or TMPRSS2 [46, 47] have been associated with high-risk PCa and may risk-stratify or prognostic individuals. Other markers associated with PCa, such as PSMA [48], may also be of interest. Future studies assessing the presence of these, or other, cell surface markers on STEAP1 positive EVs could lead to the development of a non-invasive test capable of risk-stratification or prognostication for PCa. Overall, our work highlights STEAP1 as a diagnostic marker in PCa, which can be identified on circulating EVs and provides evidence for the utility of a STEAP1 EV-based screening test to improve the clinical management of PCa.

## Supplementary information

Supplementary Data

Figure S1

Figure S2

Figure S3

Supplementary Table 1: Patient and Tumor Characteristics

Supplementary Table 2: Antibody product information
