# Supplementary material for: Clinical significance of STEAP1 extracellular vesicles in prostate cancer
Source: Prostate Cancer Prostatic Dis. 2021 Feb 15;24(3):802–11. doi: 10.1038/s41391-021-00319-2 (PMC8384631; doi:10.1038/s41391-021-00319-2)
Supplement: Supplementary file 1 — Supplementary Data [file 41391_2021_319_MOESM1_ESM.docx]

**Supplementary Data**

**Clinical Significance of STEAP1 Extracellular Vesicles in Prostate Cancer**

Karan Khanna,^1^ Nikki Salmond,^1^ Kalan S. Lynn,^2^ Hon S. Leong,^3,4^* Karla C. Williams^1^*

**Figure S1: Platelet associated EVs detected by nanoscale flow cytometry.** (A) Images are

representative of the CD9, CD41 and CD42a platelet and EV populations present in plasma as identified using nanoscale flow cytometry. (B) Quantification of the number of CD9 positive platelets and platelet derived EVs present in plasma of healthy and PCa subjects as detected by nanoscale flow cytometry. Healthy: 56 samples. PCa: 121 samples. n=2. ± SEM. Mann-Whitney test. * = P < 0.05. (C) Quantification of the number of CD41 positive platelets and platelet derived EVs present in plasma from healthy and PCa subjects as detected by nanoscale flow cytometry. Healthy: 10 samples. PCa: 10 samples. ± SEM. Mann-Whitney test and unpaired t-test. ** = P < 0.01. (D) The number of CD9 and STEAP1 positive platelets was analyzed in healthy and PCa patient plasma samples by nanoscale flow cytometry. Healthy: 56 samples. PCa: 121 samples. n=2. + SEM. Mann-Whitney test. **** = P<0.0001. (E) The number of STEAP1 events that colocalized with CD9, CD41 and CD42a EVs was quantified in healthy and PCa patient plasma samples by nanoscale flow cytometry. 10 healthy and 10 PCa whole plasma samples. ± SEM. Kruskal-Wallis ANOVA with multiple comparisons. **** = P<0.0001.

**Figure S2:** **Reproducibility of STEAP1 and CD9 quantitation by nanoscale flow cytometry.** Graphs showing the reproducibility between two different experiments using plasma samples (run 1 and run 2), and the specificity (isotype control) of nanaoscale flow cytometry. Analysis of STEAP1 (A) and CD9 (B) levels between repeats and relative to isotype control. (C) Representative images of isotype control run for STEAP1 and CD9.

**Figure S3: Total number STEAP1-postive events identified by nanoscale flow cytometry.** (A) The total number of STEAP1 positive events in 56 healthy and 121 prostate cancer plasma samples was quantified using nanoscale flow cytometry. Each sample was analyzed twice in independent repeat experiments, the average of the two experiments was used for analysis. ±SEM. Mann-Whitney test. **** = P<0.0001 (B) A receiver operating characteristic curve was generated using Graph Prism 8 to illustrate the diagnostic capabilities between STEAP1 positive events of healthy and PCa patient plasma.
