# Supplementary figures and images for: Clinical significance of STEAP1 extracellular vesicles in prostate cancer
Source: Prostate Cancer Prostatic Dis. 2021 Feb 15;24(3):802–11. doi: 10.1038/s41391-021-00319-2 (PMC8384631; doi:10.1038/s41391-021-00319-2)

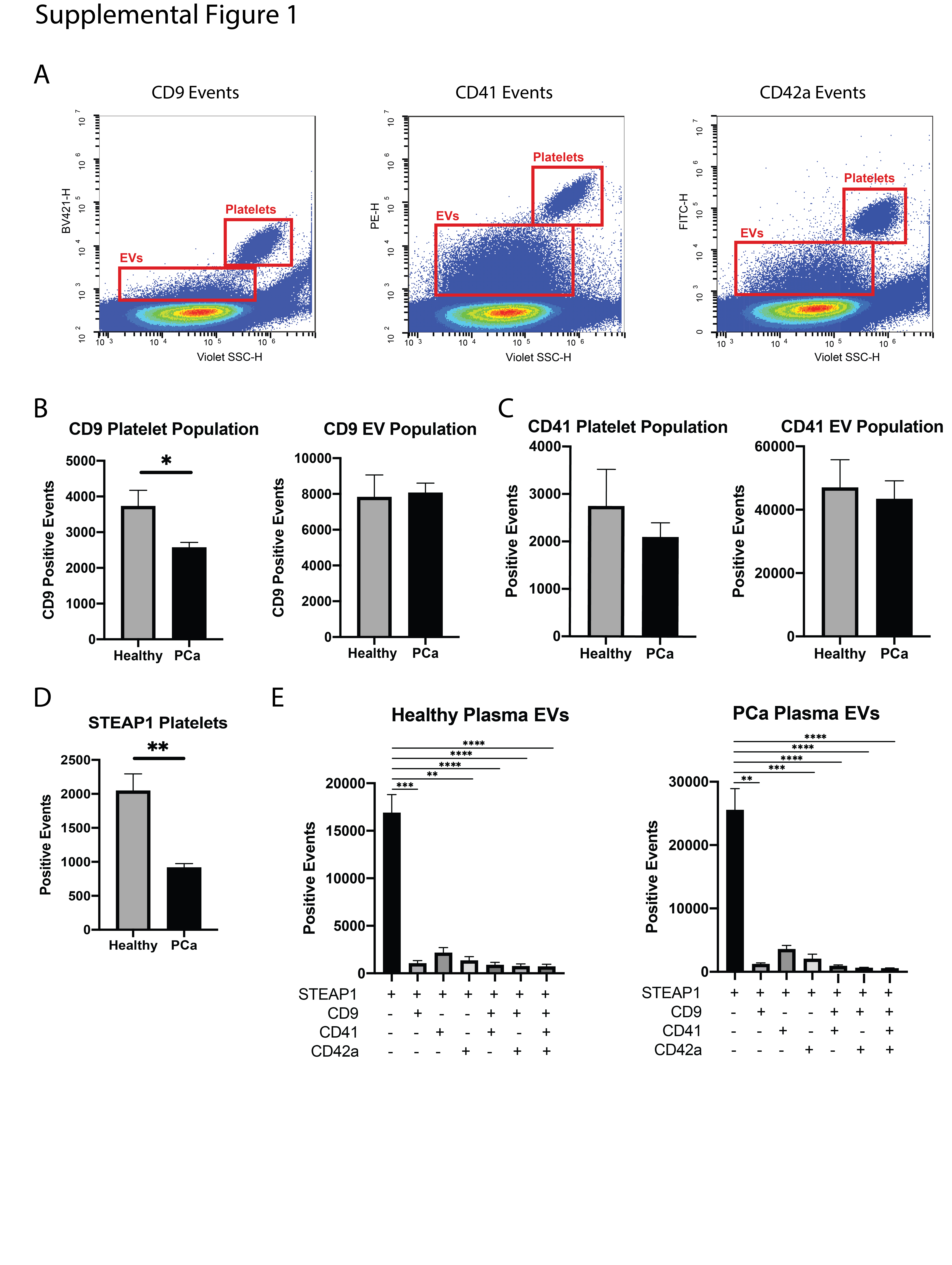

Supplement: Supplementary file 2 — Figure S1 [file 41391_2021_319_MOESM2_ESM.jpg]

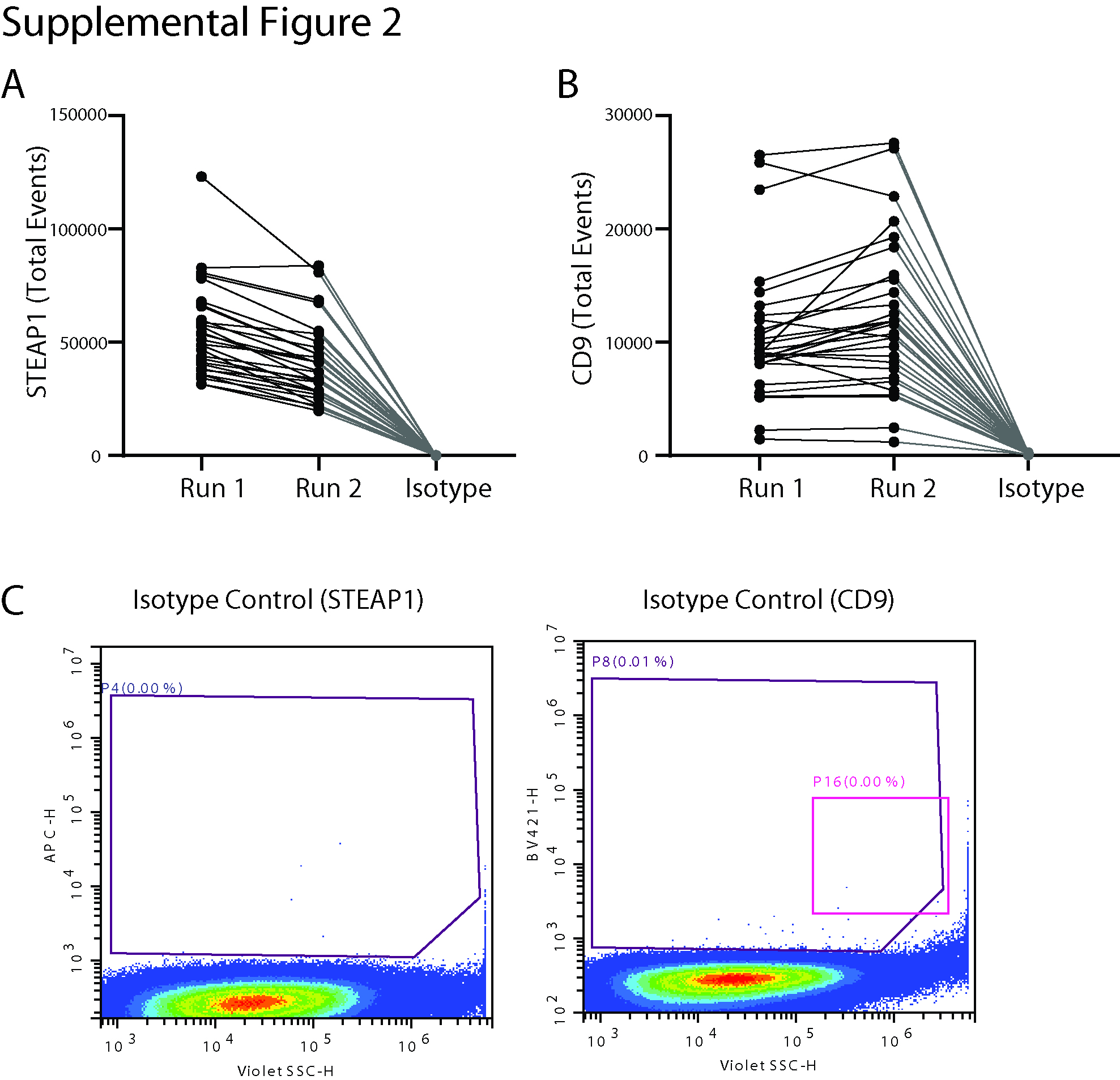

Supplement: Supplementary file 3 — Figure S2 [file 41391_2021_319_MOESM3_ESM.jpg]

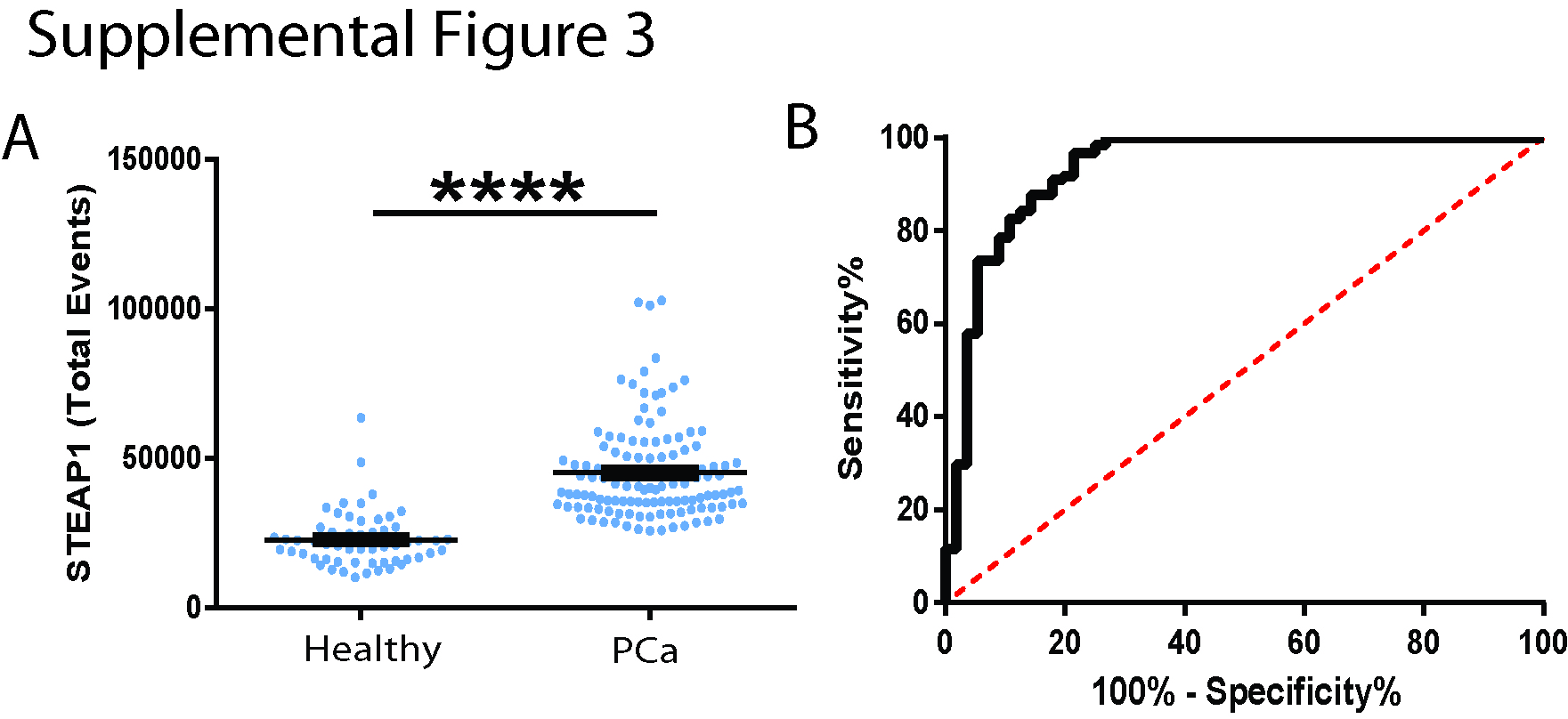

Supplement: Supplementary file 4 — Figure S3 [file 41391_2021_319_MOESM4_ESM.jpg]
